# Exploring the Ethical Landscape of Artificial Intelligence in Dentistry: Insights From a Cross-Sectional Study

**DOI:** 10.7759/cureus.82667

**Published:** 2025-04-21

**Authors:** Nadim El Khoury, Dima Hadid, Abbass El-Outa

**Affiliations:** 1 Research Ethics, Faculty of Medicine, Université Paris-Saclay, Paris, FRA; 2 Health Policy, McMaster University, Hamilton, CAN; 3 Clinical Research, Observe Group for Clinical Research, Beirut, LBN; 4 Dentistry and Oral Medicine, Private Practice, Beirut, LBN

**Keywords:** artificial intelligence, clinical decision-making, dentist-patient relationship, deontology, large language model, medical ethics

## Abstract

Background

The emergence of artificial intelligence (AI) in dentistry offers exciting prospects, alongside notable ethical hurdles. As AI capabilities advance, it is essential to comprehend the implications for dental practices, patient well-being, and the dentist-patient connection. This study aims to explore the ethical considerations and challenges associated with the use of AI in dentistry.

Methods

A web-based survey was conducted among dentists to gain a deeper understanding of their thoughts, experiences, and concerns regarding the implementation of AI in dental practice. The survey explored various aspects such as the ethical implications of AI, its effects on the relationship between dentists and patients, and the significance of human supervision in AI-assisted decision-making.

Results

The results of the study underscore the intricate ethical considerations that must be taken into account when incorporating AI technology into dental care. Dental professionals conveyed a preference for AI to serve as a supplement to human expertise, rather than a replacement, underscoring the significance of retaining human oversight and direction. Various concerns were raised regarding the potential for AI to influence clinical judgments, the importance of transparency in AI algorithms, and the necessity of safeguarding patient information. Nevertheless, the participants acknowledged the potential benefits of AI in improving diagnostic precision, treatment planning, and administrative efficacy.

Conclusion

The utilization of AI in dentistry is undoubtedly advantageous, but its implementation must be handled with care and balance. To achieve this, it is crucial to adhere to ethical standards, maintain a continuous commitment to professional education, and prioritize the preservation of the dentist-patient relationship. AI should be viewed as a tool that complements the dentist's expertise, with human judgment remaining paramount in clinical decision-making.

## Introduction

Artificial intelligence (AI) has become a game-changer in various industries, including dentistry, by promising to revolutionize diagnostic and treatment approaches [1]. AI includes fields such as machine learning (ML), deep learning (DL), and data science (DS), and aims to replicate human cognitive functions like problem-solving, perception, and language understanding [2]. While AI provides significant breakthroughs, it also presents ethical challenges, particularly in the field of dentistry, where decisions can significantly impact patient care [3].

AI technology has revolutionized medical practice by providing support in complex case decision-making processes and other areas [4]. The first FDA-approved AI-based medical imaging application was introduced in 2017, with the first AI system for detecting diabetic retinopathy being approved in 2018 - without requiring any human assistance or interpretation [5-7]. Healthcare professionals are increasingly relying on AI to provide accurate diagnoses, eliminate examiner variability, and minimize subjectivity. AI also has the potential to improve the effectiveness of care and reduce costs by eliminating certain routine examinations and tests [8], as well as by better analyzing X-ray images for more precise responses and diagnoses [9]. In this study, we will explore the various applications of AI in the field of dentistry.

AI has a significant and diverse impact on medicine and dentistry [10,11]. In medical imaging, AI algorithms enhance the accuracy of diagnoses from X-rays, MRIs, and CT scans, enabling early detection of conditions like cancer and cardiovascular diseases [10,12-14]. In dentistry, AI helps identify dental caries and periodontal diseases, leading to better patient outcomes [15]. AI also plays a vital role in treatment planning, where it can create personalized plans based on a patient's genetic profile and analyze biological data to predict drug efficacy in the development of new drugs [16]. Moreover, AI is useful in patient education and engagement, where chatbots and virtual assistants can provide information and support to improve the healthcare experience [17].

The integration of AI in healthcare presents significant ethical challenges. Concerns regarding data privacy, informed consent, and potential biases in AI algorithms must be addressed [18-20]. Moreover, utilizing AI for diagnostic and treatment decisions adds complexity to the ethical landscape, casting doubt on professional judgment, autonomy, and the importance of the human touch in patient care [11,21]. This study aims to explore the potential uses of AI in the field of dentistry, including its benefits and the ethical dilemmas that may arise. Through careful analysis of various clinical cases, the responses of AI systems, and the perspectives of dental professionals, we seek to gain a deeper understanding of how AI can support decision-making in dental practice - while also accounting for the ethical considerations involved.

## Materials and methods

Study setting and design

This is a cross-sectional study that utilized a web-based survey to explore the perceptions of dental professionals on ethical challenges associated with the use of AI in dentistry. Dentists' perspectives were then compared to responses generated with ChatGPT-4.0 (OpenAI, San Francisco, CA, USA), a state-of-the-art large language model (LLM), to evaluate its potential to guide dentists through ethical dilemmas. The study was approved by the Department of Medical Ethics at the Faculty of Medicine, Université Paris-Saclay.

Data collection

Data was collected between June and August 2023. A total of around 122 survey invitations were sent via email to a pool of dentists. Those who agreed to partake in the study were asked to click the web-based survey link, which prompted them to complete an electronic informed consent form before filling out the questionnaire. The survey was administered using the LimeSurvey platform on a dedicated server, thereby ensuring a secure and confidential environment for participants. Data was collected using various sources: social media, instant messaging groups, personalized emails, and direct contacts, to reach a broad spectrum of dental professionals. The survey was made available in English to accommodate a wider audience. Participants were assured of the confidentiality and anonymity of their responses to encourage candid answers. They were also informed that only aggregate data would be shared and disseminated.

As for the LLM, ChatGPT-4.0 used at the time was trained only up to 2021, as indicated by OpenAI. Therefore, all prompts containing information published on the web were selected exclusively from after 2022 to avoid training bias.

Measurements

The survey was comprised of three primary parts: demographics, ethical scenarios, and inquiries about AI's role in dentistry. The survey was designed to be completed within 15 minutes. The demographic inquiries encompassed age, gender, country of practice, education level, and the type of practice setting, whether private or public. Regarding knowledge of ethics in the dental office, participants were asked about common ethical practices, including whether written consent was mandatory for each patient, whether patients had the right to decide who could be informed of their medical condition, whether dentists were obligated to emphasize ethical concerns when discussing confidential information with their team members, and whether confidential patient information could be stored on personal devices. Additionally, practice attitude toward patients was evaluated, as participants were presented with a series of scenarios and asked to indicate whether they agreed or disagreed with them.

The questionnaires were constructed by a panel of four experts in the fields of dentistry, data science, and medical ethics. Questionnaire items were inspired by existing literature and expertise; some items were directly reproduced, with modifications, from existing literature, such as Razavi et al. (2023) and Alkhalifah et al. (2022) [22,23].

Knowledge and attitude indices

As previously stated, dentists' knowledge of medical and dental ethics was evaluated using a set of five questions. To analyze knowledge in a unidimensional approach, the responses were scored as 0 for incorrect answers and 1 for correct answers, with a maximum possible score of 5 and a minimum of 0. Additionally, the attitude scale, consisting of 18 questions, was used to measure ethical attitudes. Responses were rated on a scale of 1 to 4, with 4 indicating the best ethical attitude and 1 indicating the most negative attitude. The maximum score achievable on this scale was 72. The scale used is similar to the Likert scale and provides a more useful measurement of perceptions, attitudes, and opinions than a simple binary answer.

Data analysis

Data were analyzed using the R Statistical Framework 2023.03.0/RStudio 1.4.17 software (R Foundation for Statistical Computing, Vienna, Austria). Continuous variables were presented as mean and standard deviation, while categorical variables were represented by their frequency and percentage. A t-test was used to compare average knowledge and attitude scores.

## Results

Out of the 122 invitations sent, 88 responses were recorded. After data management and cleaning, 33 responses were unusable for analysis due to substantial missing data; hence, 55 full responses were retained for analysis. Table 1 presents the demographic and professional characteristics of the study participants. The majority of participants were male (n = 30, or 55%), and 25 (45%) were female, with an average age of 37 years. A significant proportion of the respondents (n = 33, or 60%) were based in Lebanon, followed by 19 (35%) in France. Three participants (5.5%) were based in other countries. The majority of participants (n = 31, or 56%) identified as specialists - predominantly in orthodontics - 21 (38%) were general practitioners, and three (5.5%) were residents currently undergoing specialization. The vast majority (n = 46, or 84%) worked exclusively in the private sector, four (7.3%) worked across both private and public sectors, and five (9.1%) practiced solely in the public sector. Moreover, 12 (22%) of the study participants were faculty members. In some sections, the number of analyzed responses dropped to 53 due to missing values.

**Table 1:** Demographic and professional characteristics of study participants

Characteristic	n (%)
Gender	
Male	30 (55%)
Female	25 (45%)
Country of practice	
Lebanon	33 (60%)
France	19 (35%)
Rest of the world	3 (5.5%)
Professional profile	
General practitioner	21 (38%)
Specialist	31 (56%)
Resident	3 (5.5%)
Primary practice setting	
Public sector	5 (9.1%)
Private practice	46 (84%)
Private and public sectors	4 (7.3%)
Faculty member	12 (22%)

A significant majority (n = 37, or 70%) of respondents acknowledged the need to obtain written consent from patients before proceeding with medical interventions, mirroring the stance recommended by AI. Conversely, 13 (25%) did not perceive this requirement as necessary, and three (5.7%) remained uncertain about the need to obtain written consent. The vast majority (n = 49, or 92%) agreed that patients should have the autonomy to decide on the disclosure of their health information, which was also supported by the AI. Only two (3.8%) believed patients lacked the right to disclose their medical condition. The majority of participants (n = 50, or 94%), supported by AI, recognized the obligation to educate their team on the importance of maintaining patient confidentiality. While 24 (45%) respondents considered it acceptable to store confidential patient information on personal devices, 20 (38%) were indecisive. The AI’s response, however, suggested that patient records should not be stored on personal devices (Table 2).

**Table 2:** Participants’ perceptions on ethical considerations (n = 53) *Denotes response chosen by ChatGPT

Question	Yes, n (%)	No, n (%)	Not sure, n (%)
Is written consent required from patients prior to dental procedures?	37 (70%)*	13 (25%)	3 (5.7%)
Do patients have the right to decide who can be informed of his/her medical condition?	49 (92%)*	2 (3.8%)	2 (3.8%)
As a dentist, do you have to educate all team members on how to maintain patient confidentiality?	50 (94%)*	1 (1.9%)	2 (3.8%)
Are you allowed to keep confidential patient information on personal devices?	24 (45%)	9 (17%)*	20 (38%)
Do patients have the right to access their dental/medical charts?	46 (87%)*	3 (5.7%)	4 (7.5%)

Ethical and clinical decision-making in dental practice

Consent and Patient Information

The majority of participants (n = 52, or 98%), supported by AI, were against prescribing unnecessary antibiotics solely to meet patient demands (disagree and strongly disagree), with only one (1.9%) of the surveyed dentists considering it acceptable to do so (Table 3).

**Table 3:** Ethical and clinical decision-making in dental practice (n = 53) *Denotes response chosen by ChatGPT

Case	Strongly disagree	Disagree	Agree	Strongly agree
A patient with very severe toothache was referred to the dentist. After examination and diagnosis, the tooth was deemed non-restorable; the dentist can take written consent from the patient and extract the tooth without explaining their problem and the ways to replace the lost tooth.	36 (68%)*	16 (30%)	1 (1.9%)	0 (0%)
A patient was referred for supragingival scaling and tooth polishing. At the end of the treatment, the dentist can prescribe antibiotics upon the patient’s request.	39 (74%)	13 (25%)*	0 (0%)	1 (1.9%)
The dentist can refuse to accept and treat LGBT (Lesbian, Gay, Bisexual, and Transgender) patients or patients with certain religions in non-emergency situations.	41 (77%)*	11 (21%)	1 (1.9%)	0 (0%)
To present the case in scientific meetings, the dentist is allowed to take several radiographs of their treatment in different directions.	11 (21%)	18 (34%)	21 (40%)*	3 (5.7%)
The dentist can receive a percentage of the patient’s fee from the radiology center in return for referring patients.	35 (66%)*	15 (28%)	3 (5.7%)	0 (0%)
A patient with very severe and annoying pain comes to your office without an appointment. After informing other patients, the dentist has to first treat the patient out of turn and then continue treating the other patients in the waiting room.	9 (17%)	20 (38%)	16 (30%)*	8 (15%)
The dentist is allowed not to inform the patient if the endodontic file is separated and remains in the root canal provided that there is no problem in finalizing the procedure.	27 (51%)*	20 (38%)	6 (11%)	0 (0%)
To persuade the patient to choose the ideal treatment, such as dental implants, the dentist can express its benefits more than other treatments, such as endodontic treatment and crown.	21 (40%)	17 (32%)*	13 (25%)	2 (3.8%)
A patient was referred to a dentist and requested that their teeth be laminated (veneers) for more beauty. The dentist can perform this treatment even though the patient is not a suitable candidate for this procedure based on scientific principles.	34 (64%)*	13 (25%)	5 (9.4%)	1 (1.9%)
You frequently see undesirable and unprincipled endodontic treatments by dentist A. In this case, you have to first inform the patients of the dentist’s error and then the given dentist, and if their treatment procedure does not change, you will have to report it to the concerned authorities.	14 (26%)	23 (43%)	16 (30%)	0 (0%)*
A patient was referred with unbearable and very severe dental pain. The dentist should explain the full process of the final treatment of choice and other alternative therapies to the patient at the same meeting before any action to obtain informed consent.	3 (5.7%)	13 (25%)	17 (32%)	20 (38%)*
For a complex problem of restoring a broken anterior tooth, the dentist refers the patient to a restorative and cosmetic specialist. Radiographs show that a number of the patient’s posterior teeth are also decayed and need treatment. The specialist can treat the mentioned tooth and other decayed teeth as well.	17 (32%)	22 (42%)	12 (23%)*	2 (3.8%)
An 8-year-old child with an avulsed central incisor presents to the dentist 15 minutes after the accident. His father informs the dentist of his financial inability to pay for the treatment. The dentist can refer the patient to more affordable centers, including colleges’ clinic and charities.	12 (23%)	24 (45%)	13 (25%)*	4 (7.5%)
The dentist is allowed not to inform the patient of the pathology (biopsy) result regarding malignancy in the oral cavity at the request of the patient’s relatives.	27 (51%)*	20 (38%)	6 (11%)	0 (0%)
A patient with pain caused by necrosis and periapical inflammation refers to a state clinic. In the first session, the dentist cleans the canals and fills them with calcium hydroxide. The dentist can persuade the patient to complete the treatment in their private office.	26 (49%)	19 (36%)*	7 (13%)	1 (1.9%)
A patient with a maxillary calcified central incisor was referred to a dentist. He tells the dentist that their tooth has changed to a yellowish color as a result of a punch in yesterday’s fight and asks him to confirm this incident. The dentist can issue a certificate for the patient to present to the judicial authorities to facilitate the complaint and receive compensation from the assailant.	8 (15%)	15 (28%)	21 (40%)*	9 (17%)
The dentist can place a crown for an infected tooth with a poor prognosis upon the patient’s request.	23 (43%)*	23 (43%)	6 (11%)	1 (1.9%)
The dentist can report a higher treatment cost at the patient’s request to receive the whole treatment cost from the insurance company.	31 (58%)*	17 (32%)	5 (9.4%)	0 (0%)

Diagnostic Imaging

While 24 (45.7%) participants supported the use of multiple X-rays for better case documentation, 11 (21%) and 18 (34%) responded with strongly disagree and disagree, respectively, regarding the use of excessive radiography. The AI's response endorsed the use of multiple X-rays for scientific purposes (Table 3).

Financial Incentives and Emergency Care

A minority (n = 3, or 5.7%) found it acceptable for dentists to receive a percentage of radiology center fees for referring patients, a stance the AI strongly disagrees with. In emergencies, such as severe pain, the decision to prioritize treatment outside of scheduled appointments was evenly split among respondents (Table 3).

Disclosure of Procedure Complications

Notably, six (11%) of participants believed it was unnecessary to inform patients about damaged and retained endodontic instruments if the procedure was otherwise completed successfully, a view that was opposed by the AI (Table 3).

Aesthetic Treatments and Professional Ethics

Around a tenth of participants would honour the patient's request, while 47 (89%) participants advocated for adherence to scientific principles - a stance also supported by the AI. The handling of sub-optimal treatments performed by peers was controversial; 37 (69%) would refrain from discussing another dentist's errors with patients, prioritizing professional relationships and patient confidence. However, 16 (30%) tended to inform the patient, confront the colleague, and possibly report to authorities if changes were not made, which aligns with the AI's recommendation (Table 3).

Specialist Referrals and Financial Hardship

Regarding complicated cases requiring specialist referral, such as a broken anterior tooth, opinions differed; 39 (74%) participants reported being against treating non-referred issues during the referral visit, whereas 12 (23%) considered the treatment acceptable, which aligns with the AI’s response. Moreover, 17 (32.5%) practitioners supported referring patients in emergency settings to more affordable care options in cases of financial challenge, which also aligns with the AI’s response (Table 3).

Compromised Treatment Requests

Importantly, 46 (86%) participants were against placing a crown on an infected tooth with a poor prognosis at the patient’s request - a stance also supported by the AI (Table 3).

We also explored dental professionals' familiarity and engagement with AI in their practice. Only 28 (52%) respondents felt well-acquainted with dental AI, with a mere eight (16%) participants incorporating AI into their daily operations. Interestingly, four (6%) were uncertain about using AI-based software in their practices. The majority (n = 38, or 71%) of participants believed that the main advantage of AI is the automation of routine tasks to save time. Additionally, 31 (59%) recognized AI's potential to enhance diagnostic precision and effectiveness, while 29 (54%) saw its value in improving treatment and care planning. Notably, 18 (34%) participants believed that AI grants access to a broader range of dental skills.

Only 33 (62%) participants expressed concern over the potential biases and impacts of AI algorithms on care outcomes, with 7 (13%) unconcerned and 13 (25%) undecided. Furthermore, 31 (58%) of dentists felt ill-equipped to tackle the ethical challenges AI introduces to dentistry; nevertheless, 46 (86%) showed interest in training programs on its ethical application. About 25 (48%) practitioners believed AI would influence the dentist-patient relationship; 64% foresaw both positive and negative effects, 13 (24%) predicted solely negative consequences, and six (12%) anticipated exclusively positive outcomes.

A fraction of 12 (22%) of practitioners consider AI a useful tool for ethical decision-making, providing objective, data-driven insights. However, 21 (39%) doubted AI's utility in such sensitive matters, attributing this skepticism to the nuanced, emotional, and contextual aspects of ethical issues that AI might not fully grasp, and 22 (41%) reported being unsure of AI's role in ethical decision-making. Regarding liability for medical errors involving AI, there is a consensus among dentists that the ultimate responsibility should not lie with AI software, with only three (6%) attributing blame to AI.

**Figure 1:**
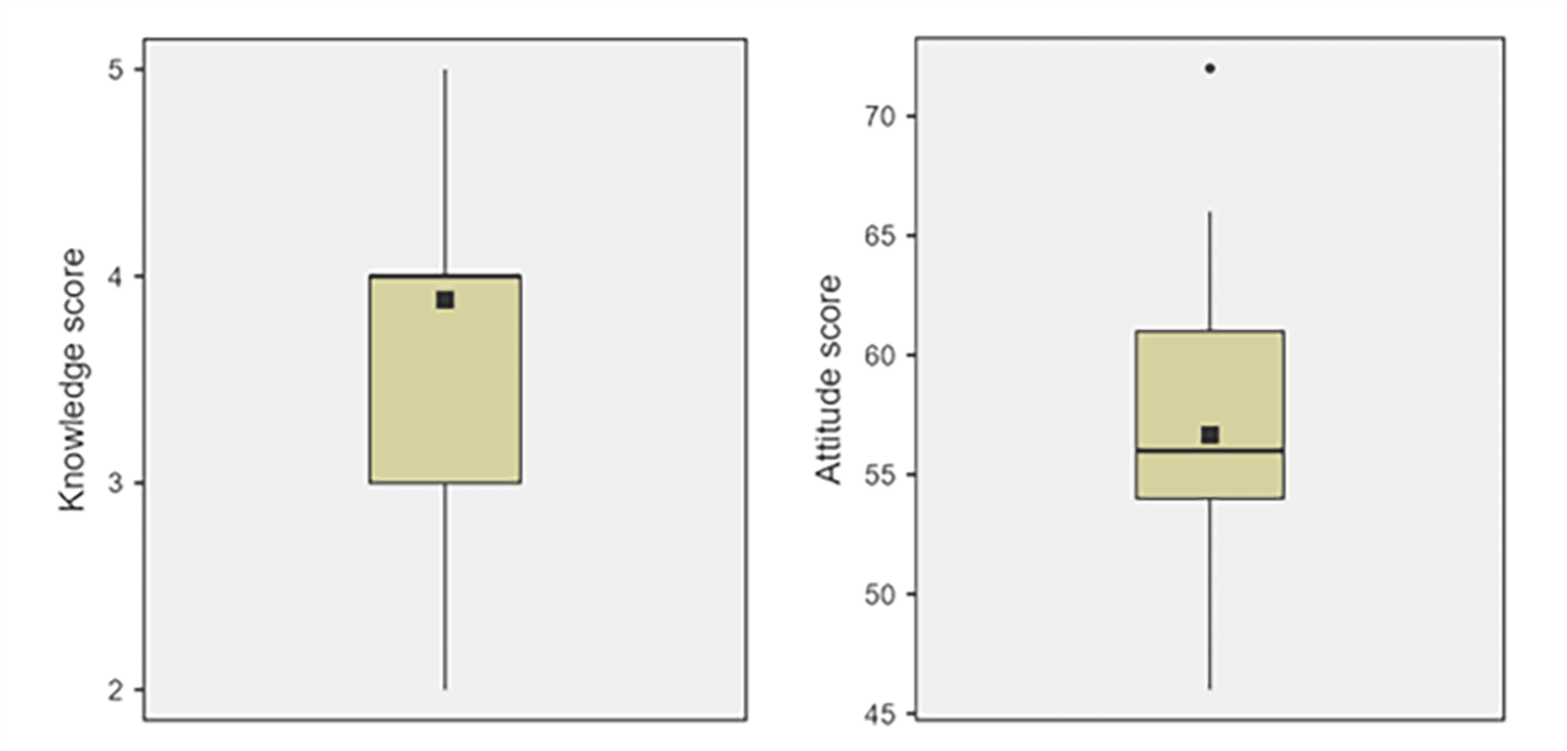
Box plots of knowledge and attitude scores of participating dentists Horizontal line: median; Black square: mean; Black dot: outlier

Analysis of the knowledge index revealed that ChatGPT-4 achieved a score of 5, which demonstrates a robust understanding of ethical guidelines and decision-making frameworks in dentistry (Table 4). This score was significantly higher than the average score of 3.9 achieved by human participants, indicating that the AI has a strong understanding of the technical and ethical knowledge necessary for practice (Figure 1). On the other hand, attitude scores were different (Table 4). Human participants scored higher on the attitude index, with an average of 56.7, compared to the score of 53 achieved by ChatGPT-4, despite not being statistically significant. This highlights the point that human dentists may have more nuanced, empathetic approaches that are pivotal in ethical dental practice.

**Table 4:** Knowledge and attitude indices characteristics

Statistic	Knowledge index	Attitude index
Average (std dev)	3.9 (0.8)	56.7 (5.3)
Range	2.0 - 5.0	46.0 - 72.0
AI score	5	53
p	<0.0001	0.999

## Discussion

The introduction of AI in dentistry has opened up a diverse range of opportunities and ethical dilemmas. This research has highlighted the intricacy of incorporating AI into dental practice, emphasizing the significance of a well-balanced approach that neither dismisses AI altogether nor overestimates its abilities. Our findings revealed that there is a consensus on the crucial role of human oversight in AI-assisted decision-making. This reflects a wider concern about the possibility of AI operating independently, without taking into account the essential clinical expertise of dentists.

Although AI has the potential to improve diagnoses and treatment planning, dentists prefer its use as a supplementary tool rather than a substitute for human judgment. This preference highlights the invaluable worth of professional expertise and the nuanced understanding of patient care that AI is currently unable to replicate [24]. Our findings also shed light on a crucial element of medical practice: informed consent. The AI’s adherence to the ethical and medical guidelines of informed consent underscores the importance of engaging patients in their care choices. This adherence not only upholds ethical norms but also cultivates a relationship of trust between dentists and patients, ensuring that care decisions are made with the patient’s informed consent.

Another important ethical consideration that we can infer from this study is the safeguarding of patient information. The unanimous agreement between AI-generated responses and dental experts on the importance of maintaining medical confidentiality highlights the ethical duty to uphold patient privacy. This responsibility is particularly crucial, given the delicate nature of health-related data and the possible consequences of its unauthorized exposure [23]. Medical confidentiality is considered a central tenet of the healthcare system, as it plays a vital role in protecting the privacy and autonomy of patients [25]. Information pertaining to an individual's health is highly personal and must be treated with the utmost care and confidentiality, except in situations where the law permits or mandates the disclosure of medical information [25]. The principle of medical confidentiality is not only a legal obligation but also an ethical one, reflecting the importance of respecting the privacy, autonomy, and dignity of patients [25].

To uphold the principle of medical confidentiality, healthcare providers are expected to maintain a high level of professionalism and discretion in handling patients' medical information [26,27]. This includes ensuring that confidential information is only disclosed on a need-to-know basis to authorized individuals, such as other healthcare providers involved in the patient's care [25]. Healthcare providers are also obligated to take adequate measures to safeguard patients' medical information against unauthorized access, theft, or misuse [25]. Failure to comply with the principle of medical confidentiality can result in serious legal and ethical repercussions, including litigation, disciplinary action, and loss of professional reputation [25]. Furthermore, it can have a detrimental impact on the trust and confidence of patients in the healthcare system, which is critical for the effective delivery of healthcare services [28]. As such, healthcare providers must remain vigilant in upholding the principle of medical confidentiality, while also balancing the competing interests of patient privacy and public safety.

Our findings revealed a unanimous agreement that patients have the right to be fully informed about their diagnoses and treatment options, highlighting the ethical responsibility of transparency in dental practice [29]. The Code of Ethics laid down by the American Dental Association underscores the significance of patient autonomy and informed consent, which aligns with our investigation, highlighting the necessity of transparency and honesty in patient communication [30].

Additionally, the ethical concerns surrounding financial incentives in patient referrals necessitate a re-evaluation of practices that could potentially compromise the objectivity of clinical decision-making. It is imperative to underscore that the practice of offering a commission or a percentage of the fees to a dentist in exchange for referring patients to a radiology center is fraught with ethical concerns and can pose significant problems [31]. While this approach may have potential drawbacks, there are valid concerns regarding the dentist's impartiality in directing patients to radiology centers [31]. There is a risk that personal financial gain may sway the dentist's decision-making, rather than prioritizing the patient’s actual needs [31,32]. Additionally, there may be a temptation to over-prescribe radiological tests to increase profits from the radiology center [31,32]. Such behavior could be seen as a violation of ethical standards in the dental profession, which prioritize the patient’s best interests and require the dentist to provide optimal care without being influenced by financial motives [31,32]. It is of utmost importance that medical decisions, such as guiding patients toward radiology services, are grounded in sound clinical practices, medical ethics, and the patient’s best interests [31,32]. Should a radiology center appoint a dentist to establish a comparable practice, AI can serve as an impartial source of information, offering unbiased guidance to promote the highest quality and most ethical medical practices.

Moreover, our findings regarding the use of antibiotics and X-rays further highlight the ethical complexities of integrating AI into dental practice. These findings serve as a reminder of the importance of prioritizing medical necessity and ethical considerations when making clinical decisions, rather than simply adhering to routine practice or succumbing to patient demands [33]. It is crucial to understand that the prescription of antibiotics for oral health purposes must be based on a thorough assessment of the patient’s medical background and relevant considerations [33]. Antibiotics are not routinely prescribed after every dental cleaning and polishing, as this can contribute to antibiotic overuse, leading to antibiotic resistance and other health complications. If a patient has specific concerns or requests regarding antibiotics or any other medication, it is essential to have an open discussion with their dentist. The dentist will consider the patient’s medical history, oral health, and other relevant factors before deciding to prescribe antibiotics. Dentists are healthcare professionals who follow established protocols and best practices to ensure patient safety and treatment effectiveness [34]. Responsible medical decision-making is crucial for optimal dental care and long-term patient health [35]. Therefore, it is important to note that medication should not be prescribed to a patient unless their medical situation requires it, and the decision should be made by a qualified medical professional. Moreover, it is imperative to inform the patient of the reasons behind any additional X-rays and to secure their informed consent before proceeding with the examination [36]. While multiple X-rays may be necessary to document a case accurately, it is crucial to approach this in a responsible and medically justified manner, taking into account the patient’s ethical and safety concerns [37]. Effective communication and collaboration between the dentist and the patient are critical in making informed decisions about the use of X-rays [36].

Our research supports and expands upon existing literature regarding the integration of AI in healthcare. Our findings concur with existing literature that views AI as a tool for automating routine tasks, improving diagnostic and treatment outcomes, and accessing a broader range of dental skills [8,37]. However, these studies also share our concerns regarding the ethical implications of AI, including issues of responsibility, data privacy, bias, and the impact on the dentist-patient relationship. Another study by Smith et al. (2024) on the use of AI in medical diagnostics found that healthcare professionals hold a cautious optimism regarding AI, stressing the need for it to complement, rather than replace, human expertise [37]. Our study concurs with these findings and indicates that dentists perceive AI as a supportive tool and recognize the importance of professional judgment in clinical decision-making. Additionally, our findings concur with those of Patel et al. (2022), which identified ethical concerns surrounding patient privacy and data security regarding the use of AI [38]. We also emphasize the significance of informed consent and transparency in dental practice, which is in line with Zhang et al.'s (2021) argument that patient autonomy and participation are critical in the age of AI [39]. However, our study offers unique insights into the specific challenges and opportunities presented by AI in dentistry, highlighting the nuanced understanding of patient care and the ethical implications of technology adoption [40]. Comparing our findings to those of other healthcare disciplines, it is evident that while ethical challenges are common, the context of dental practice provides a distinct perspective on how to navigate these challenges for the benefit of patient care.

The tension between adhering to a strict schedule and responding to acute patient needs, even at the expense of disrupting scheduled appointments, highlights the importance of professional ethics and patient care [41]. Clear communication and transparency with all patients involved are essential in such situations, as they demand a delicate balance, guided by the dentist's ethical duty to alleviate suffering and the practical considerations of clinic management [41].

Our survey revealed another ethical issue related to the nondisclosure of endodontic instrument breakage during treatment, with a small fraction of dentists believing in withholding such information from patients. This practice raises significant ethical concerns, as the principle of informed consent and the obligation to disclose any complications during treatment are foundational to medical ethics and patient trust [42]. The majority stance, supported by AI, advocates for transparency and honesty, reinforcing the ethical imperative to inform patients about all aspects of their care, including potential complications.

Additionally, the survey highlights the importance of ethical considerations surrounding patient requests for unnecessary or unsuitable treatments. The overwhelming consensus among dentists and AI, against acquiescing to patient demands that contravene scientific principles, underscores the importance of professional judgment and ethical responsibility in dental practice [43]. This finding highlights the importance of dentists acting in the best interest of their patients, guided by evidence-based practice and ethical standards, rather than yielding to patient requests that may not be in their best health interest [43].

One issue that arose during the survey was professional integrity and how to handle perceived substandard care by colleagues. It seems that many dentists are hesitant to directly confront or report questionable decisions made by their peers. This is likely due to a complex interplay of professional solidarity, patient trust, and ethical accountability. Dentists often prefer indirect communication strategies when discussing treatment options with colleagues, in order to maintain professional relationships while still prioritizing patient care [43].

Additionally, the survey also revealed that dentists have concerns about the role of AI in ethical decision-making and clinical practice. While there is interest in the potential of AI to support clinical decisions, there is also significant skepticism about its ability to navigate the complex ethical landscapes of dental practice [43]. As a result, many dentists are cautious about integrating AI recommendations into their practice, and emphasize the irreplaceable value of human judgment and the ethical complexities inherent in dental care.

This disparity between the knowledge and attitude index scores provides valuable insights into the role of AI in dentistry. While ChatGPT-4's perfect score on the knowledge index underscores its potential as a powerful tool for navigating complex ethical guidelines, its lower score on the attitude index, compared to human dentists, emphasizes a significant limitation: the inability of AI to fully replicate the empathetic and nuanced ethical reasoning that is essential in patient care. These findings suggest that although AI can enhance the technical aspects of dental practice, the human element remains irreplaceable. Human dentists not only apply ethical knowledge but also interpret and adjust it according to the emotional and moral contexts of each patient interaction. Therefore, it is crucial that AI is used as an adjunct tool that supports, but does not replace, the dentist’s judgment, ensuring that patient care is both technically sound and compassionately delivered [44].

Strengths and limitations

The strengths of our study are depicted in several aspects. First, to our knowledge, this study is among the very few that compare medical ethics knowledge and attitudes between a commercial, widespread AI model and human experts. Since AI is being integrated more and more in medicine and dentistry, such studies are warranted to better understand the use and limitations of this integration. Second, a crucial point in AI-related clinical studies that deserves highlighting is the training bias of the AI model. The model performs better when facing data already included in its training. Hence, in our study, we made sure the literature used to develop the questionnaire was more recent than the date of ChatGPT 4.0's initial training. Therefore, ChatGPT 4.0 was blind to the specific questions that were adapted from existing literature.

Our study should be considered in light of its limitations. Our study focused on the integration and impact of AI in dental practice. While our sample included practitioners from diverse backgrounds with varying levels of experience and practice locations, we observed a notable geographic concentration, with over 60% of respondents practicing in Lebanon, where the adoption of AI in dental clinics may not be as advanced as in other regions, such as Europe or the United States. This geographical skew limits the generalizability of our findings, as responses from practitioners in countries with more widespread use of AI could have provided additional insights into its integration and impact on dental practice. Moreover, our study did not include interviews with dental students, both those in specialized programs and those yet to obtain their dental degrees, which represents a missed opportunity to explore students' concerns and expectations regarding AI's place in their field and education. Engaging with this demographic could have enriched our understanding of AI's role and perception within the academic sphere, offering a forward-looking perspective on how AI is shaping the future of dental education and practice. Lastly, we did not survey patients, a significant limitation, considering their central role in the dental care ecosystem. Patient feedback on their experiences with AI in dental settings and its perceived impact on the dentist-patient relationship would have added valuable dimensions to our findings. Understanding patient perspectives could have provided a more holistic view of AI's integration into dental care and its implications for patient satisfaction and trust.

Future recommendations 

Given the high knowledge of AI models, areas like empathy and a positive attitude in practice need further scrutiny. Future studies may delve deeper into studying the necessary ethical guidelines for the integration of AI in clinical practice, as well as establishing frameworks for the cooperation of human-AI experts. Ultimately, the aim is to improve the patient experience with positive outcomes.

## Conclusions

This study underscores the nuanced interplay between AI and the field of dentistry, revealing both the promising potential and the ethical complexities of integrating AI into dental practice. While AI offers significant advantages in enhancing diagnostic accuracy, treatment efficiency, and administrative processes, it also introduces challenges that necessitate careful consideration and management. The findings emphasize the indispensable role of human oversight in AI-assisted decision-making, highlighting the irreplaceable value of empathy, patient rapport, and professional judgment that define the essence of dental care. Dentists recognize the need for a balanced approach that leverages AI as a supportive tool, rather than a replacement for human expertise. This balance is crucial for preserving the integrity of the dentist-patient relationship and ensuring that care remains patient-centered. The study calls for the development of robust ethical guidelines, continuous professional education on AI's capabilities and limitations, and collaborative efforts to establish standards ensuring AI's transparent, accountable, and secure use in dentistry. Ultimately, AI should be embraced as a complement to, not a substitute for, the skilled and compassionate care provided by dental professionals.
